# Risky injection practices and HCV awareness in Chiang Mai Province, Thailand: a respondent-driven sampling study of people who inject drugs

**DOI:** 10.1186/s12889-020-09549-w

**Published:** 2020-09-24

**Authors:** Myrtille Prouté, Sophie Le Coeur, Métrey H. Tiv, Timothée Dub, Parinya Jongpaijitsakul, Anantika Ratnamhin, Chaisiri Angkurawaranon, Apinun Aramrattana, Marc Lallemant

**Affiliations:** 1 French Institute for Demographic Studies (INED), Mortality, Health and Epidemiology unit, 9 cours des Humanités, CS 50004, 93322 Aubervilliers, France; 2 French National Research Institute for Sustainable Development (IRD), IRD174/PHPT, 195 Kaew Nawarat Road (3-4 Fl.) Wat Ked, Muang Chiang Mai, 50000 Thailand; 3 Faculty of Medicine, Department of Family Medicine, Chiang Mai University, Chiang Mai, Thailand

**Keywords:** People who inject drugs PWID, HCV, Respondent-driven sampling RDS, Thailand

## Abstract

**Background:**

People who inject drugs (PWID) are the most exposed to hepatitis C virus (HCV). In Thailand, drug use is highly criminalized, and harm reduction services are scarce. This study estimates risky injection practices and assesses the proportion of HCV awareness and screening in the PWID population in Northern Thailand.

**Methods:**

We used respondent-driven sampling (RDS) to recruit PWID in Chiang Mai Province. Social and behavioural data were collected through face-to-face interviews at an addiction treatment facility. Weighted population estimates were calculated to limit biases related to the non-random sampling method. Univariate and multivariate analyses were performed to study factors associated with HCV awareness and screening.

**Results:**

One hundred seventy-one PWID were recruited between April 2016 and January 2017. Median age was 33 (Interquartile range: 26–40) years, 12.2% were women, and 49.4% belonged to a minority ethnic group. Among participants, 76.8% injected heroin, 20.7% methadone, and 20.7% methamphetamine. We estimate that 22.1% [95% CI: 15.7–28.6] of the population had shared needles in the last 6 months and that 32.0% [95% CI: 23.6–40.4] had shared injection material. Only 26.6% [95% CI: 17.6–35.6] had heard of HCV. Factors independently associated with knowledge of HCV included belonging to a harm reduction organization (adjusted odds ratio (aOR) = 5.5 [95% CI: 2.0–15.3]) and voluntary participation in a drug rehabilitation programme (aOR = 4.3 [95% CI: 1.3–13.9]), while Lahu ethnicity was negatively associated (aOR = 0.3 [95% CI: 0.1–0.9]). We estimate that 5% of the PWID population were screened for HCV; the only factor independently associated with being screened was membership of a harm reduction organization (aOR = 5.7 [95% CI: 1.6–19.9]).

**Conclusion:**

Our study reveals that the PWID population is poorly informed and rarely screened for HCV, despite widespread risky injection practices. A public health approach aimed at reducing the incidence of HCV should target the PWID population and combine harm reduction measures with information and destigmatization campaigns. Civil society organizations working with PWID are a major asset for the success of such an approach, based on their current positive interventions promoting awareness of and screening for HCV.

## Background

Seventy-one million people, or 1% of the world population, were estimated to be living with chronic hepatitis C virus (HCV) infection in 2015 [1]. In 2016, the World Health Assembly adopted a global health sector strategy to combat viral hepatitis, which included eliminating hepatitis C by 2030 [2]. This strategy followed the advent of direct-acting antivirals (DAAs), molecules offering a fast, safe, and highly effective cure for people infected with HCV [3], but only a small fraction have benefited from the treatment so far [4]. Even at an affordable cost [5], treatment availability is insufficient to eliminate HCV [6].

Chiang Mai is the largest city in northern Thailand, close to the ‘Golden Triangle’, the area where the borders of Thailand, Myanmar and Laos meet. It is known as one of the world’s largest opium-producing areas and an entry route for heroin and methamphetamine [7]. This mountainous region is home to several “hill tribes”, ethnic minorities living in isolated villages. Use of heroin and opium is more prevalent than in other Thai provinces where methamphetamine use is predominant [8].

While the safety around blood transfusions and invasive medical procedures has improved significantly, injecting drug use remains the main factor fuelling the HCV epidemic in many countries [1]. The prevalence of HCV infection among people who inject drugs (PWID) ranges from 65% to more than 80% globally [9]. In high-income countries, 50–80% of new HCV infections are found among PWID [10]. Yet despite being the population most affected by HCV—and the existence of recommendations for the assessment, management, and treatment of HCV among PWID—their access to screening and treatment remains excessively limited worldwide [11].

Following the World Health Organization’s publication of a regional action plan for viral hepatitis in South-East Asia [12], the Thai Ministry of Public Health has issued a strategic plan for HCV prevention and control. Its main goal is to increase treatment coverage and decrease transmission via prevention [13]. Although the strategy acknowledges the high HCV prevalence in PWID, its main prevention recommendations focus on blood donation safety measures and on encouraging PWID to seek addiction treatment. Thailand has recently received a voluntary licence for generic DAAs from Gilead Sciences. Scaling up DAAs at an affordable price within the Thai strong public health system, which already proved efficient in tackling the HIV epidemic [14], could bring the country to the forefront of HCV elimination. It is in PWID that treatment and prevention can have the greatest impact on the spread of the epidemic. However, health policies targeted towards this particularly hard-to-reach population might be at odds with law enforcement policies. Since chronic HCV infection is usually silent during the first decades of infection, most infected patients are asymptomatic and may not know their HCV status. Additionally, widespread prejudice towards PWID may limit access to information and screening. The complex HCV care pathway in Thailand makes it difficult for PWID to access diagnosis and treatment, as only liver specialists are authorized to deliver these treatments and have little experience in dealing with PWID specificities. On the other hand, MOUD (medication for opioid use disorder) is prescribed by addiction specialists who usually do not venture into the field of infectious diseases.

The prevalence of HCV in the general population in Thailand is estimated, based on scant data, at 1–3% [15–17], representing around 350,000 individuals with active HCV infection [18]. Several studies have identified injection drug use as the main risk factor of new infection [19, 20]. The estimated number of PWID ranges from 40,300 to 160,500 individuals in Thailand [21–23] and from 3000 to 4000 individuals in Chiang Mai [24], with estimated HCV prevalence ranging from 44 to 86% [25–27]. This extremely high HCV prevalence among PWID concurs with global and regional estimates [28]. The few studies estimating HCV screening uptake among PWID in Thailand found low rates coupled with low awareness of the disease [29–31], suggesting that most PWID infected with HCV do not know their status. This lack of awareness is correlated with difficulties in accessing healthcare generally [32].

In Thailand, the use of recreational drugs is illegal, and repression is the dominant societal response [33]. As a result, PWID are frequently arrested and incarcerated for drug-related offences [34]. Public health interventions such as needle exchange programmes, MOUD prescriptions, and health education have proven effective in reducing HIV and viral hepatitis transmission by more than 50% among PWID, provided these measures are made widely available [35–37]. Several studies have reported high prevalence of risky injection practices among Thai PWID, such as syringe borrowing, that could be prevented with appropriate harm reduction programmes [30, 38, 39]. Some harm reduction interventions, such as access to MOUD with methadone and naloxone, have been successfully implemented [40]. However, these medications are unavailable in prisons, and frequent incarceration of PWID has been documented as the main barrier to retention in methadone maintenance therapy [41, 42], along with police interference [43] and lack of accessibility in rural areas [44]. Needle exchange programmes remain confined to pilot projects reliant on international donors [40].

Most studies among PWID in Thailand have used convenience-based sampling methods, recruiting participants from harm reduction organizations or enrolled in clinical trial, and were conducted in Bangkok [45–47]. Few epidemiological studies have used methods such as respondent-driven sampling to obtain data from a representative sample of PWID [8, 48]. However, these studies did not collect information on HCV, which is a major infectious risk in this population.

The knowledge of the HCV treatment cascade among PWID is crucial for evaluating the progress towards HCV elimination. Information on their risk of reinfection due to risky injection practices and on their access to the healthcare system will help target interventions to end HCV transmission in this population. This study was carried out to inform the national strategy on the specificities of the PWID population regarding HCV. We estimate risky injection practices and assess the proportion of HCV awareness and screening and their associated factors in the PWID population in Northern Thailand.

## Methods

### Formative assessment

Before the survey, we conducted a formative assessment of HCV prevention and treatment practices focusing on PWID. We carried out in-depth interviews with key informants: PWID, members of harm reduction organizations, and healthcare professionals specialized in drug addiction and liver diseases. The results were used to develop the study protocol: recruitment sites, preferred time to access participants, reasonable amount of financial compensation, and variables to include in the survey questionnaire.

### Study population and participant selection

This study focused on the PWID population of Chiang Mai Province, Thailand. Researchers consider PWID a hard-to-reach population due to their comparatively small number, their reluctance in view of the stigma and illicitness of their activity, and the absence of a sampling frame [49]. We selected participants who met the following inclusion criteria: aged 18 and older, living in Chiang Mai Province, and having injected any drug in the last 6 months. We used respondent-driven sampling (RDS) to recruit participants. This form of chain-referral sampling was designed for hard-to-reach populations [50, 51]. RDS uses a system of structured compensation with quotas (weights). Each participant is invited to recruit a fixed and limited number of peers from his or her social network, and this limited number reduces biases seen in other chain-referral methods.

While RDS has been widely used in the last two decades and adopted by leading public health organizations [52, 53], the quality of estimates derived from these data has been challenged [54, 55]. To mitigate such concerns, we monitored potential sources of participation bias by asking participants about their motivation to participate in the study and if they had difficulties giving out coupons when they came to collect their compensation. To account for bias linked to finite population effects, we used the estimator based on successive sampling [56].

The initial group of participants was selected purposively and recruited for interviews; these ‘seeds’ were then asked to recruit and refer peers. Peers then presented a coupon, proof of their recruitment by a peer. Each PWID could participate only once. Interviews and referrals of peers formed recruitment waves. We chose the seeds from PWID networks identified during the formative assessment in order to represent the diversity in age, sex, ethnicity, and drug used among the PWID population in Chiang Mai Province.

### Study procedures

We carried out participant recruitment and interviews in locations where participants would feel safe and where the local police would not drop by. These locations were identified during the formative assessment and based on experience of staff members involved in harm reduction organizations.

Seeds participants were given three recruitment coupons to recruit peers who met the inclusion criteria. Recruited peers were in turn asked to recruit three others and so on. In compliance with the RDS method, participants received a primary incentive of THB (Thai bahts) 200 (USD 6.4) for their participation and a secondary incentive of THB 50 (USD 1.6) for each peer recruited. The initial aim was to recruit up to 300 participants to estimate key parameters with sufficient precision. A sample size of 300 is needed to detect a population proportion of 30% in a population size of 3000 with a 95% confidence level and a 5% margin of error. This figure was based on the estimated population size derived from the formative assessment and the literature [20, 24, 48].

Participants underwent an inclusion interview with a study staff member to confirm eligibility. The inclusion interview was assigned to a single study staff member, who would pay attention to and remember the participants throughout the study to prevent multiple participation. If doubts about injection drug usage arose, an interviewer experienced with PWID would ask questions on drugs and drug use (effects and modes of consumption) and would look for evidence of injection marks. Participants were included after their written informed consent was signed.

### Questionnaire

We carried out a face-to-face interview with each participant in Thai or in other local languages with an interpreter—using a structured, quantitative questionnaire. The questionnaire was developed for the purposes of this study, using information from the formative assessment and templates from questionnaires used in similar studies developed by the authors. The questionnaire was pilot tested with PWID for acceptability before study launch. Besides sociodemographic characteristics, the questionnaire explored living circumstances, past and current drug use, injection practices, awareness and knowledge of HCV (symptoms, risk factors, transmission routes, screening and treatment history), access to healthcare services and drug addiction care, experience with the healthcare system, and history of incarceration or police custody. We provide the English version of the questionnaire as an Additional file 1 with permission of the authors.

### Data analyses

Descriptive data are first reported with their unweighted sample size and percentages, then weighted percentages account for the RDS method, using the successive sampling estimator [56]. The estimated proportions in the population after adjustment are presented in the tables and referred to as “population estimates”. Only raw sample proportions are presented in the text. Statistical analyses included the successful seeds.

To study the factors associated with HCV knowledge and screening, we used generalized linear models (log-binomial regression). Exposure variables were first tested in univariate analysis. Then all variables associated with the outcomes with a *p-value* < 0.2 were included in the multivariate analysis using forward selection. Age, sex, and ethnicity variables were forced into the multivariate models due to their potential as confounding factors. Multivariate analyses were performed with unweighted data. The results are reported as adjusted odd ratios (aOR) with their 95% confidence interval (CI).

Analyses were performed using Stata version 13.0 and RDS Analyst 0.5–2 for the RDS weighted estimates. Recruitment chains in Fig. 2 were drawn using NetDraw 2.158.

## Results

### RDS seed participants and survey flow

**Fig. 1:**
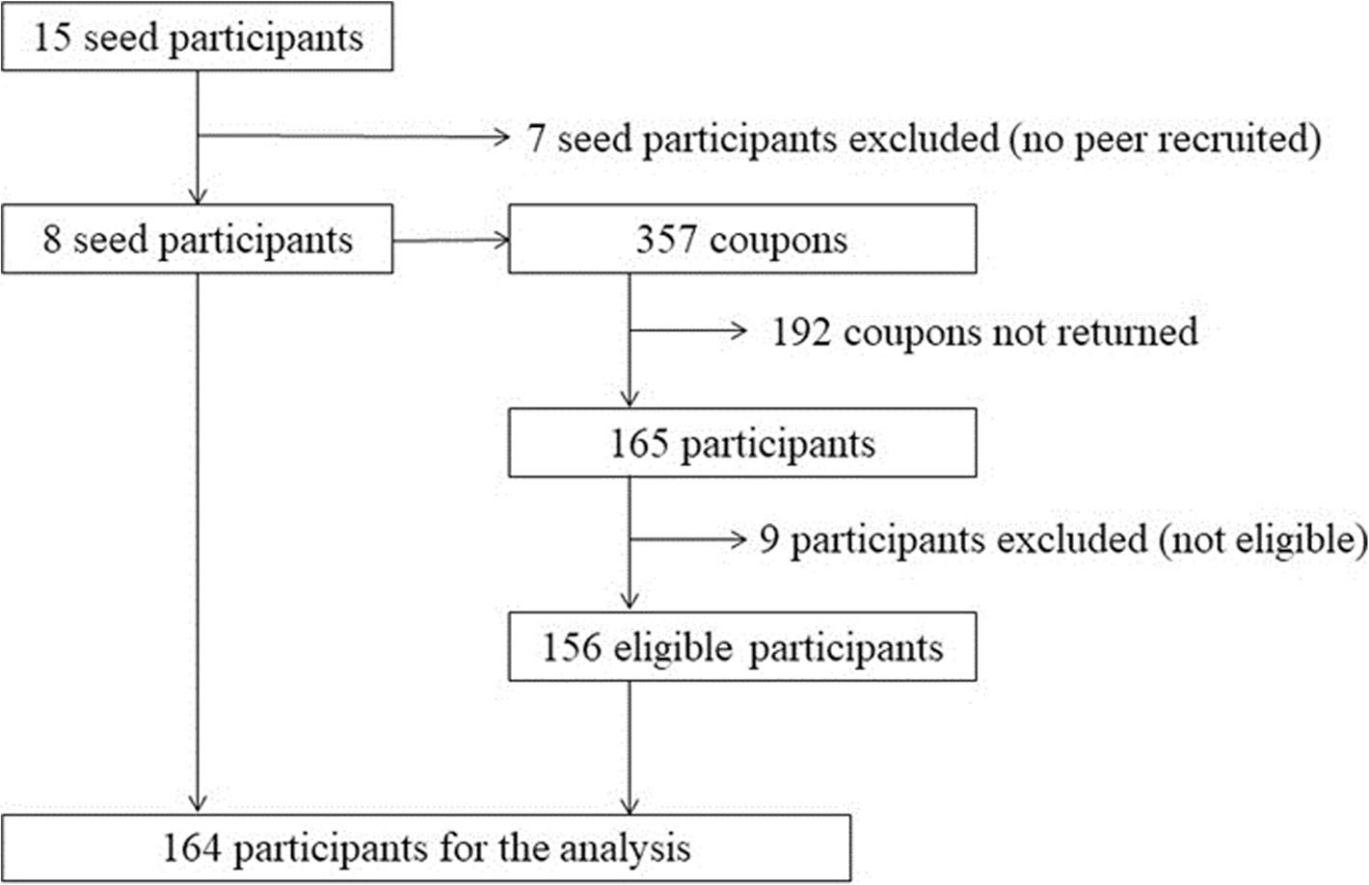
Flowchart of participants

The study took place from April 2016 to January 2017 at the Fah Mai Clinic, a public healthcare facility in the city of Chiang Mai offering care for addiction, and on appointment in other locations according to participants’ preferences. We started the recruitment with eight seeds; seven more were added later to address a slowdown in recruitment. In total, 15 seeds were recruited, of which 7 failed to recruit other participants and were excluded from the study, leaving a final number of 8 seed participants. We distributed 357 recruitment coupons, of which 165 were returned (46%), leading to the recruitment of 156 eligible participants (94%) by 8 seed participants (Fig. 1). The longest recruitment chain comprised 11 waves. Equilibrium was reached from the 6th wave for the main variables (sex, ethnicity, and HCV knowledge).

**Fig. 2:**
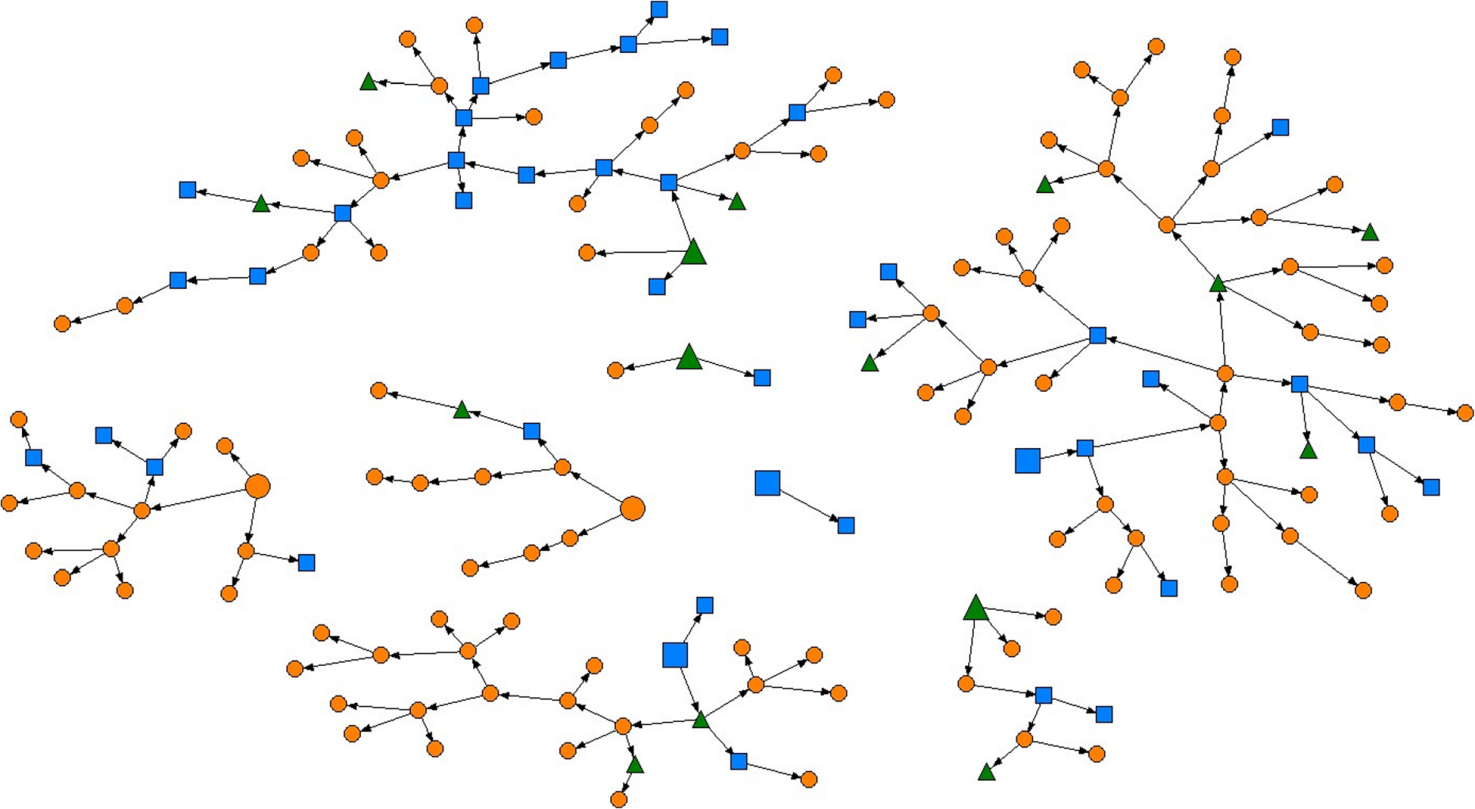
Recruitment chains by HCV knowledge and screening. Each element represents a participant in the survey. Arrows point from recruiters to recruits. Seeds are represented with bigger elements; orange circles represent participants who had never heard of HCV before the survey; blue squares represent those who had heard of HCV; and green triangles represent those who had already been tested for HCV

Figure 2 represents the recruitment chain of each seed. The seeds had a mean age of 29 years, 25% were female, 63% were of Thai ethnicity, 75% had heard of HCV, and 38% had been screened for it.

### Sociodemographic characteristics

Table 1 presents the sociodemographic characteristics of survey participants. Median age was 33 years [IQR: 26–40]; 12% were female; 51% were ethnically Thai, 26% Lahu, and 24% from other ethnic minorities (Akha, Lisu, Tai Yai, Burmese, and Karens); 16% did not have Thai nationality. Median weekly income was THB 500 [IQR: 300–7400]. Seventy-six per cent of the participants worked informally, had a temporary day job, or had no income. Sixty per cent had already been in prison (police custody was not considered).

**Table 1:** Sociodemographic characteristics

Sociodemographic characteristics (***N*** = 164)	***N*** (%)	Population estimates in % [95% CI]
Sex (female)	20 (12.2)	12.3 [6.5–18.0]
Age (years)		
< 25	32 (19.5)	16.5 [8.0–25.1]
25–34	59 (36.0)	35.4 [25.6–45.2]
35–44	46 (28.1)	28.1 [19.3–36.8]
≥ 45	27 (16.5)	20.0 [10.2–29.8]
Ethnic group		
Thai	83 (50.6)	46.1 [32.9–59.2]
Lahu	42 (25.6)	34.7 [21.2–48.4]
Other^a^	39 (23.8)	19.2 [10.9–27.4]
Nationality (Thai)	138 (84.2)	83.1 [75.4–90.9]
Family situation		
In a relationship/married	79 (48.2)	52.8 [42.6–63.0]
Has dependents (children or relatives)	99 (60.4)	61.0 [51.5–70.5]
Education (high school or higher)	70 (42.7)	36.9 [25.8–48.0]
Activity		
Employed	31 (18.9)	19.1 [11.4–26.8]
Independent worker	9 (5.5)	6.0 [1.0–11.1]
Informal work/daily work	108 (65.9)	64.1 [52.3–76.0]
No activity/student	16 (9.8)	10.7 [0.0–22.1]
Weekly income in THB^b^		
< 1000	16 (9.8)	12.7 [6.2–19.3]
1000–2500	90 (54.9)	57.3 [48.7–66.0]
2500–4000	43 (26.2)	23.6 [16.0–31.1]
≥ 4000	15 (9.2)	6.3 [2.5–10.2]
Ever been arrested for drug use	114 (70.0)	68.1 [59.0–77.3]
Ever been in prison	99 (60.4)	56.8 [46.7–67.2]

^a^Akha, Lisu, Tai Yai, Burmese, and Karens

^b^USD 1.00 = THB 31.20 (Thai baht), conversion rate at the time of the study

### Access to the healthcare system and to drug addiction care

Table 2, on access to health- and drug-addiction care, reveals that 11% had no health insurance, 7% had been denied care, and 16% had faced discrimination in healthcare settings due to their drug use. Sixty per cent had enrolled in a programme in a rehabilitation centre, including 25% in a compulsory programme; 67% of the participants had received methadone substitution treatment. Twenty-four per cent were members of a harm reduction organization.

**Table 2:** Access to the healthcare system and to drug addiction care

Access to the healthcare system (***N*** = 164)	***N*** (%)	Population estimates in % [95% CI]
Does not have health insurance	18 (11.0)	11.7 [5.4–18.0]
Ever been refused care	11 (6.7)	5.2 [1.5–8.8]
Faced discrimination in a healthcare setting due to drug use	26 (15.9)	12.7 [6.5–19.0]
Received care for addiction	123 (75.5)	76.2 [65.9–86.5]
Current or past treatment with methadone	110 (67.1)	69.3 [53.3–85.5]
Participated in a voluntary programme in a rehabilitation centre	97 (60.3)	61.1 [49.5–72.6]
Ever been in a compulsory rehabilitation centre	40 (24.5)	23.7 [14.9–32.5]
Member of a harm reduction organization	38 (23.5)	17.8 [11.0–24.7]

### Drug use and risky injection practices

Table 3 shows that the main drugs used were heroin (79%), methamphetamine (45%) and sedatives, such as midazolam and other sleeping pills (31%). Methadone was used by 70%. Twenty-six per cent had experienced at least one overdose. More than one third (34%) reported injecting drugs daily in the past month. Although 88% were getting needles from a pharmacy, only 12% were getting them from a needle exchange programme. Finally, 28% per cent reported sharing their syringes in the past 6 months, and 33% reported sharing other injection material (containers, water, filter).

**Table 3:** Recent drug use and injection practices

Drug use and injection practices (***N*** = 164)	***N*** (%)	Population estimates in % [95% CI]
Drug used in the last 6 months		
Heroin	130 (79.3)	84.5 [77.1–92.0]
Methamphetamine	74 (45.1)	42.6 [29.7–55.2]
Sedatives	51 (31.1)	26.7 [16.8–36.5]
Opium	28 (17.1)	17.8 [9.0–26.6]
Methadone	115 (70.1)	72.1 [54.6–89.7]
Age at first intravenous drug use (years)		
< 18	31 (18.9)	16.0 [9.2–23.0]
18–24	63 (38.4)	38.1 [27.9–48.2]
≥ 25	70 (42.7)	45.9 [35.2–56.6]
Duration of intravenous drug use since first injection (years)		
< 1	25 (15.2)	15.3 [8.2–22.2]
1–5	49 (29.9)	31.9 [22.3–41.3]
5–10	34 (20.7)	16.4 [10.0–23.0]
≥ 10	56 (34.2)	36.4 [27.4–45.7]
Frequency of injection (mutually exclusive)		
Every day	55 (33.6)	29.5 [19.0–40.0]
Several times per week, but less than every day	31 (18.9)	16.0 [9.5–22.5]
Several times per month, but less than every week	21 (12.8)	13.3 [5.8–20.7]
Once a month or less	57 (34.8)	41.1 [30.8–51.7]
Needle supplier		
Pharmacy	145 (88.4)	87.9 [81.3–94.4]
Needle exchange programme	20 (12.2)	8.0 [3.4–12.5]
Friends	22 (13.4)	16.9 [9.8–24.0]
Shared needles (last 6 months)	45 (27.6)	22.1 [15.7–28.6]
Shared other injection material (last 6 months)	54 (32.9)	32.0 [23.6–40.4]
Reused needles (last 6 months)	109 (66.5)	63.1 [52.2–73.9]
Ever experienced an overdose	43 (26.2)	22.6 [15.0–30.3]

### HCV knowledge

Thirty-four per cent of the participants (*n* = 56) reported they had heard of HCV before the study; the corresponding weighted population estimate was 26.6% [95% CI: 17.6–35.6]. Among these participants, 73% mentioned that the virus can be transmitted through blood or needle sharing, but only 23% could mention accurate hepatitis C symptoms.

The multivariate analysis (Table 4) showed that knowledge of HCV was independently associated with membership of a harm reduction organization (aOR = 5.5, *p* = 0.001), and previous voluntary participation in a drug rehabilitation programme (aOR = 4.3, *p* = 0.015), while Lahu ethnicity was negatively associated (aOR = 0.3, *p* = 0.038). However, after adjustment, education and history of incarceration did not remain significantly associated with HCV knowledge.

**Table 4:** Analysis of factors associated with HCV knowledge

Variables	Univariate analysis		Multivariate analysis	
	OR [95% CI]	***p*** value	aOR [95% CI]	***p*** value
Sex (female)	0.8 [0.3–2.2]	0.677	0.8 [0.2–4.2]	0.797
Age (years)				
< 25	–	–	–	–
25–34	1.4 [0.6–3.6]	0.470	0.8 [0.2–2.5]	0.650
35–44	1.7 [0.7–4.7]	0.235	0.9 [0.3–3.1]	0.915
≥ 45	0.9 [0.3–2.8]	0.850	0.4 [0.1–2.2]	0.296
Ethnic group				
Thai	–	–	–	–
Lahu	0.3 [0.1–0.6]	0.006	0.3 [0.1–0.9]	0.038
Other^a^	0.8 [0.3–1.7]	0.510	1.3 [0.5–3.7]	0.604
Education (high school or more)	2.2 [1.1–4.2]	0.019		
Drug use				
Shared needles	1.3 [0.6–2.6]	0.501		
Shared other injection material	1.2 [0.6–2.4]	0.585		
Drug addiction treatment				
Methadone	1.1 [0.4–3.2]	0.801		
Member of a harm reduction organization	5.1 [2.4–11.2]	0.000	5.5 [2.0–15.3]	0.001
Voluntary participation in drug rehabilitation	3.0 [1.0–8.5]	0.043	4.3 [1.3–13.9]	0.015
Compulsory participation in drug rehabilitation	1.2 [0.6–2.5]	0.630		
Ever been in prison	2.0 [1.0–4.1]	0.045		
Ever been denied care	1.1 [0.3–3.9]	0.872		
Discriminated against due to drug use	2.2 [0.9–5.1]	0.075		

^a^ Akha, Lisu, Tai Yai, Burmese, and Karens

### HCV screening

Of the 56 participants who had heard of HCV infection before the study, 15 reported having previously been tested for HCV (9.1% of all 164 participants); the weighted population estimate was 5.2% [95% CI: 2.7–7.8]. Only five had been tested in the last 12 months. Four people among the 15 screened tested positive, and treatment had been proposed to one person.

As Table 5 shows, the only factor independently associated with HCV screening was membership of a harm reduction organization (aOR = 5.7, *p* = 0.007).

**Table 5:** Analysis of factors associated with HCV screening

Variables	Univariate analysis		Multivariable analysis	
	OR [95% CI]	***p*** value	aOR [95% CI]	***p*** value
Sex (female)	0.5 [0.1–3.9]	0.501	0.6 [0.1–5.9]	0.629
Age (years)				
< 25	–	–	–	–
25–34	1.1 [0.2–6.3]	0.923	1.1 [0.2–7.3]	0.902
35–44	3.2 [0.6–16.0]	0.165	2.9 [0.5–17.2]	0.246
≥ 45	0.6 [0.0–6.7]	0.661	0.9 [0.1–12.7]	0.925
Ethnic group				
Thai	–	–	–	–
Lahu	0.2 [0.0–1.3]	0.084	0.4 [0.0–4.0]	0.425
Other^a^	0.5 [0.1–2.1]	0.375	1.2 [0.2–5.6]	0.855
Education (high school or higher)	6.3 [1.7–23.2]	0.006	3.7 [0.8–17.6]	0.103
Drug use				
Shared needles	0.4 [0.1–1.9]	0.257		
Shared other injection material	1.4 [0.5–4.2]	0.542		
Drug addiction treatment				
Methadone	0.9 [0.2–4.6]	0.944		
Member of a harm reduction organization	8.5 [2.7–26.8]	0.000	5.7 [1.6–19.9]	0.007
Voluntary participation in drug rehabilitation	1.5 [0.3–7.3]	0.618		
Compulsory participation in drug rehabilitation	0.8 [0.2–2.8]	0.669		
Ever been in prison	1.3 [0.4–4.1]	0.622		
Ever been denied care	1.0 [0.1–8.3]	0.995		
Discriminated against due to drug use	2.1 [0.6–7.1]	0.248		

^a^Akha, Lisu, Tai Yai, Burmese, and Karens

## Discussion

Our survey reveals that the PWID population in Chiang Mai Province is poorly informed about HCV and is at high risk of infection, while access to health services is still limited. We estimate that only one quarter of the PWID population have heard of HCV and that only 5% have been screened. At the same time, one fifth have shared syringes and one-third other injection material in the last 6 months.

Several barriers to healthcare were reported in the survey. Nearly half of the participants were ethnic minorities, 17% of whom did not have Thai nationality and therefore had limited access to the health system. Membership of an ethnic minority was a factor negatively associated with HCV awareness. Five per cent reported they had experienced denial of care, and 13% had faced discrimination in healthcare settings due to their drug use. For fear of discrimination, members of this population fail to report their addiction, which in turn prevents them from accessing counselling and testing for blood-borne diseases, including HCV. These results are consistent with those of a study conducted among PWID in Bangkok which found that healthcare avoidance was associated with care refusal and other barriers to access [32].

We suggest that measures be taken to remove these barriers, particularly for ethnic minorities who do not have Thai nationality and are consequently denied access to universal health coverage. Our concerns over the highly restrictive criteria for granting HCV therapy have been raised with key informants. In Thailand, health professionals often require patients to stop consuming recreational drugs 6 months before starting treatment. This prerequisite excludes PWID from accessing HCV treatment.

The high prevalence of risky injection practices found in our study reflects the lack of harm reduction services and easy access to clean syringes and needles. High prevalence of syringe lending has been documented in Bangkok and Chiang Mai [30, 38, 39]. However, needle sharing in Chiang Mai seems to be more frequent than in Bangkok where PWID are more exposed to harm reduction interventions [8].

We have shown that compulsory rehabilitation treatment in a detention centre has no influence on HCV knowledge or testing, whereas such treatment carried out voluntarily is positively associated with HCV knowledge. We suggest that access to treatment in rehabilitation centres should be open to PWID willing to participate and that prevention information on infectious diseases in these centres should be provided.

As with other studies conducted in Thailand [29, 30, 57], we found very low HCV awareness and screening uptake among PWID in Chiang Mai. However, at the time of our study, PWID seemed to have confidence in health centres providing methadone. Therefore, we also suggest that health professionals in these centres be trained to provide information on infectious risks linked with injection drug use and that they refer their patients for screening and treatment. Health institutions, such as centres delivering MOUD or drug rehabilitation services, may be effective intermediaries for reaching and informing PWID. Integrating and decentralizing services, especially in the fields of addictions and infectious diseases, along with increased involvement of primary health care [18], might be necessary to simplify access to harm reduction interventions and to HCV diagnosis and treatment in PWID [57].

One study among male PWID in Southern Thailand has evaluated the uptake of HCV testing at 39.5% [29], another among PWID in Bangkok at 33% [31], and yet another among HIV-positive PWID in Bangkok at 52.2% [30]. Compared to our results, these higher rates may be due to more contacts with harm reduction organizations where HCV testing is promoted. In our study, the only factor associated with HCV screening was membership of a harm reduction organization. The local network of non-governmental organizations (NGOs) and health volunteers supporting the PWID population in Chiang Mai Province is active and respected. Their positive impact on the level of HCV knowledge and screening could be strengthened to fight the epidemic.

### Limitations to the study

Over the course of our quantitative survey, we encountered several difficulties related to PWID, a hard-to-reach population. First, the expected sample size could not be reached for different possible reasons: the size of the PWID population in Chiang Mai Province may be smaller than previously estimated; also, we may have reached a threshold of participants, beyond which stigmatization, fear of being arrested, and cost of transportation prevented more participants from coming to the study site. This latter hypothesis seemed to be confirmed after recruiting participants by appointments in different sites: a Buddhist temple in the surrounding area of Chiang Mai—temples are considered safe from police interventions—and a village in the mountains outside the city. Additionally, our study excluded PWID under age 18 for ethical reasons, but this group does account for a sizeable portion of the target population according to the data collected from key informants during the formative assessment.

Chiang Mai Province is large and PWID who live in remote areas, especially in mountain villages, were more difficult to reach; this more isolated subgroup is likely to have less access to health information than urban PWID. People working at night or in the morning were probably under-represented in the survey, as few people took up the offer to participate by appointment outside the Fai Mai Clinic’s office hours.

Most participants spoke Thai, except a few people from minority ethnic groups; for these individuals, we agreed that a family member or friend could accompany the participant and act as an interpreter. This practice may have lowered the reliability of their answers.

Our study, along with others conducted in Thailand [8, 48], shows that it is possible to conduct RDS surveys among PWID in the Thai context. This type of survey could be done regularly for surveillance in this population, to measure the HCV burden and monitor the treatment cascade accurately. A major obstacle to the conduct of the survey was the current repression and stigmatization towards drug users in Thailand. The transition of the country’s response to drug use, from repression to a harm reduction policy, would facilitate broader studies of this hard-to-reach population, thus leading to more effective interventions.

## Conclusions

Membership of a harm reduction organization was the single most important predictor for HCV knowledge and screening in PWID. This finding calls for extending harm reduction interventions, including in prison settings, by expanding access to MOUD throughout the country, introducing community-wide needle and syringe exchange programmes, and providing targeted information about the risks associated with HCV. Thailand’s political will to invest in reducing the price of HCV treatment; the positive signals sent by the government to transition towards a harm reduction policy; the effective network of NGOs—an essential asset for reaching drug users—all give hope that we can control the HCV epidemic in this population.

## Supplementary information

**Additional file 1.**

## Data Availability

The data that support the findings of this study are available from the corresponding author upon reasonable request. The data are not publicly available due to their containing information that could compromise participant protection and privacy.

## Abbreviations

DAAs: Direct-Acting Antivirals
HCV: Hepatitis C Virus
NGO: Non-Governmental Organization
MOUD: Medication for Opioid Use Disorder
PWID: People Who Inject Drugs
RDS: Respondent-Driven Sampling
