# Supplementary material for: Risky injection practices and HCV awareness in Chiang Mai Province, Thailand: a respondent-driven sampling study of people who inject drugs
Source: BMC Public Health. 2020 Sep 24;20:1450. doi: 10.1186/s12889-020-09549-w (PMC7517806; doi:10.1186/s12889-020-09549-w)
Supplement: Supplementary file 1 — Additional file 1. [file 12889_2020_9549_MOESM1_ESM.pdf]

RDS Screening Form

Coupon number I__I__I__I__I__I__I__I__I__I__I__I__I__I__I__I__I__I	
Interviewer initials _ _ _ _ _ _	Date of interview I__I__I I__I__I I__I__I__I__I<br>Time interview start I__I__I H I__I__I M
1. How old are you?	I__I__I years old<br>❒ **>= 18, eligible** ❒ **<18, ineligible**
2. When was the last time you injected drugs?<br>(– ask to see stigmata)	_ _<br>❒ **< 6 months, eligible** ❒ **> 6 months, ineligible**
3. In what province do you live or work – or socialize?	_ _<br>❒ **Chiang Mai Province, eligible** ❒ **Other province, ineligible**
4. What is the main reason you accepted a coupon and enrolled in this survey?<br>***(Select only one response)***	❒1 For the incentive<br>❒2 Recommendation from person who gave coupon<br>❒3 The survey seemed interesting/useful<br>❒4 I have free time<br>❒77 Other (Specify): _<br>_ _<br>_ _<br>_ _
Conduct consent form now	
5. How many people do you know, (you know their name and they know yours), who have injected drugs in the past six months?<br>***(Participants must provide a response to this question – response cannot be zero)***	**Number** I__I__I__I__I
6. How many of them have you seen in the past month?<br>**(*Participants must provide a response to this question – response cannot be zero*)**	**Number** I__I__I__I__I<br>*(this number cannot be larger than number above)*
7. How many of them are 18 years and older?<br>**(*Participants must provide a response to this question – response cannot be zero*)**	**Number** I__I__I__I__I<br>*(this number cannot be larger than number above)*
8. How many of them live in the Chiang Mai Province?<br>**(*Participants must provide a response to this question – response cannot be zero*)**	**Number** I__I__I__I__I ***-> Report number on the checklist***<br>*(this number cannot be larger than number above)*
9. How many of them are female?<br>**(*Participants must provide a response to this question*)**	**Number** I__I__I__I__I<br>*(this number cannot be larger than number above)*

Interviewer's notice:

- ***Do not read answers.***
- ***Show the question's corresponding card when mentioned.***
- ***Ask for specifications when mentioned.***
- ***Skip questions when mentioned.***

**SECTION 1 SOCIAL DEMOGRAPHIC CHARACTERISTICS**		
"I would like to start this interview by asking you questions about your personal background."		
No.	QUESTION	ANSWER
1.1	What is your gender?	❒1 Male<br>❒2 Female<br>❒3 Transgender<br>*❒88 DNK ❒99 Refusal*
1.2	What year were you born?	I_2_I_5_I__I__I<br>*❒88 DNK ❒99 Refusal*
1.3	What is your ethnicity?	_ _ _ _ _ _ _ _ _ _ _ _ _ _ _ _ _ _ _ _<br>*❒88 DNK ❒99 Refusal*
1.4	Do you have the Thai citizenship?	❒1 No<br>❒2 Yes (specify color of ID card): _<br>*❒88 DNK ❒99 Refusal*
1.5	What is your marital status?	❒1 Single (never married)<br>❒2 Married<br>❒3 In a relationship<br>❒4 Widowed<br>❒5 Divorced or separated<br>❒77 Other (Specify): _<br>_ _<br>*❒88 DNK ❒99 Refusal*
1.6	Do you have children or relatives that you are supporting?	❒1 No<br>❒2 Yes (Specify): _<br>*❒88 DNK ❒99 Refusal*
1.7	What is your highest education level?	❒1 I didn't go to school<br>❒2 I didn't finish primary school<br>❒3 Primary school<br>❒4 Secondary school<br>❒5 Some vocational, teacher training<br>❒6 College or university<br>❒77 Other (Specify): _<br>*❒88 DNK ❒99 Refusal*
1.8	Which language do you feel most comfortable using?	Language _<br>*❒88 DNK ❒99 Refusal*

1.9	What is your Thai language ability?<br>a) Understand<br>b) Speak<br>c) Read<br>d) Write	 <br>❐1 ☺ ❐2 😐 ❐3 ☹ *❐88 DNK ❐99 Refusal*<br>❐1 ☺ ❐2 😐 ❐3 ☹ *❐88 DNK ❐99 Refusal*<br>❐1 ☺ ❐2 😐 ❐3 ☹ *❐88 DNK ❐99 Refusal*<br>❐1 ☺ ❐2 😐 ❐3 ☹ *❐88 DNK ❐99 Refusal*
1.10	What are your current living circumstances (in the last month)?	❐1 In your own place<br>❐2 At your family's place<br>❐3 At a friend's place<br>❐4 In an institution<br>❐5 In daily rental place<br>❐6 On the street<br>❐77 Other (Specify): ___________________________________<br>____<br>*❐88 DNK ❐99 Refusal*
1.11	In the place(s) you are currently staying, is there:<br>a) Piped water?<br>b) Electricity?<br>c) Toilets?	 <br>❐1 No ❐2 Yes *❐88 DNK ❐99 Refusal*<br>❐1 No ❐2 Yes *❐88 DNK ❐99 Refusal*<br>❐1 No ❐2 Yes *❐88 DNK ❐99 Refusal*
1.12	Do you have access to internet?	❐1 No ➔ 1.14<br>❐2 Yes<br>*❐88 DNK ❐99 Refusal*
1.13	Where do you connect to the internet usually?<br>***(Show the card, several answers possible)***	❐1 Own computer<br>❐2 Own mobile phone<br>❐3 Friend's computer<br>❐4 Friend's mobile phone<br>❐5 Internet café<br>❐77 Other (Specify): ___________________________________<br>____<br>*❐88 DNK ❐99 Refusal*
1.14	What is your current working status?	❐1 Permanent wage work<br>❐2 Government employee<br>❐3 Day labor<br>❐4 Student<br>❐5 Unemployed<br>❐6 Unable to work<br>❐77 Other (Specify): ___________________________________<br>____<br>*❐88 DNK ❐99 Refusal*
1.15	Do you have a regular income?	❐1 No<br>❐2 Yes<br>*❐88 DNK ❐99 Refusal*

1.16	What are your main sources of income?<br>*(Several answers possible)*	❒1 Employment<br>❒2 Daily wages<br>❒3 Support from NGOs<br>❒4 Support from government<br>❒5 Support from family<br>❒6 Selling things<br>❒7 Selling sex<br>❒8 None<br>❒77 Other (Specify): _<br>*❒88 DNK ❒99 Refusal*
1.17	What is your income approximately?	_ _ _ _ _ _ _ _ _ _ _ _ Baht<br>❒1 Per day<br>❒2 Per week<br>❒3 Per month<br>*❒88 DNK ❒99 Refusal*

SECTION 2 DRUG USE

"The next questions are related to the practice of using drugs."

Question	Heroin	Opium	Methadone	Ya-Ba	Ice	Midazolam/ sleeping pill	Cannabis	Glue	Cocaine	Ecstasy	Alcohol	Other (Specify): ________ ________
2.1 Which drugs have you used in the last 6 months? ***(Several answers possible)***	❐1 No<br>❐2 Yes<br>❐88 DNK<br>❐99 Refusal	❐1 No<br>❐2 Yes<br>❐88 DNK<br>❐99 Refusal	❐1 No<br>❐2 Yes<br>❐88 DNK<br>❐99 Refusal	❐1 No<br>❐2 Yes<br>❐88 DNK<br>❐99 Refusal	❐1 No<br>❐2 Yes<br>❐88 DNK<br>❐99 Refusal	❐1 No<br>❐2 Yes<br>❐88 DNK<br>❐99 Refusal	❐1 No<br>❐2 Yes<br>❐88 DNK<br>❐99 Refusal	❐1 No<br>❐2 Yes<br>❐88 DNK<br>❐99 Refusal	❐1 No<br>❐2 Yes<br>❐88 DNK<br>❐99 Refusal	❐1 No<br>❐2 Yes<br>❐88 DNK<br>❐99 Refusal	❐1 No<br>❐2 Yes<br>❐88 DNK<br>❐99 Refusal	❐88 DNK<br>❐99 Refusal
(For each drug used in the last 6 months, ask the questions below in a vertical order)												
2.2 How often did you use it?<br>1) One or several times per day<br>2) One or several times per week but not every day<br>3) Several times per month but not every week<br>4) Less than that	↓<br>❐1<br>❐2<br>❐3<br>❐4<br>❐88 DNK<br>❐99 Refusal	↓<br>❐1<br>❐2<br>❐3<br>❐4<br>❐88 DNK<br>❐99 Refusal	↓<br>❐1<br>❐2<br>❐3<br>❐4<br>❐88 DNK<br>❐99 Refusal	↓<br>❐1<br>❐2<br>❐3<br>❐4<br>❐88 DNK<br>❐99 Refusal	↓<br>❐1<br>❐2<br>❐3<br>❐4<br>❐88 DNK<br>❐99 Refusal	↓<br>❐1<br>❐2<br>❐3<br>❐4<br>❐88 DNK<br>❐99 Refusal	↓<br>❐1<br>❐2<br>❐3<br>❐4<br>❐88 DNK<br>❐99 Refusal	↓<br>❐1<br>❐2<br>❐3<br>❐4<br>❐88 DNK<br>❐99 Refusal	↓<br>❐1<br>❐2<br>❐3<br>❐4<br>❐88 DNK<br>❐99 Refusal	↓<br>❐1<br>❐2<br>❐3<br>❐4<br>❐88 DNK<br>❐99 Refusal	↓<br>❐1<br>❐2<br>❐3<br>❐4<br>❐88 DNK<br>❐99 Refusal	↓<br>❐1<br>❐2<br>❐3<br>❐4<br>❐88 DNK<br>❐99 Refusal
2.3 How do you currently use it? ***(Several answers possible)***<br>1) Oral<br>2) Sniffed<br>3) Smoked<br>4) Inhaled<br>5) Injected	❐1<br>❐2<br>❐3<br>❐4<br>❐5<br>❐88 DNK<br>❐99 Refusal	❐1<br>❐2<br>❐3<br>❐4<br>❐5<br>❐88 DNK<br>❐99 Refusal	❐1<br>❐2<br>❐3<br>❐4<br>❐5<br>❐88 DNK<br>❐99 Refusal	❐1<br>❐2<br>❐3<br>❐4<br>❐5<br>❐88 DNK<br>❐99 Refusal	❐1<br>❐2<br>❐3<br>❐4<br>❐5<br>❐88 DNK<br>❐99 Refusal	❐1<br>❐2<br>❐3<br>❐4<br>❐5<br>❐88 DNK<br>❐99 Refusal	❐1<br>❐2<br>❐3<br>❐4<br>❐5<br>❐88 DNK<br>❐99 Refusal	❐1<br>❐2<br>❐3<br>❐4<br>❐5<br>❐88 DNK<br>❐99 Refusal	❐1<br>❐2<br>❐3<br>❐4<br>❐5<br>❐88 DNK<br>❐99 Refusal	❐1<br>❐2<br>❐3<br>❐4<br>❐5<br>❐88 DNK<br>❐99 Refusal	❐1<br>❐2<br>❐3<br>❐4<br>❐5<br>❐88 DNK<br>❐99 Refusal	❐1<br>❐2<br>❐3<br>❐4<br>❐5<br>❐88 DNK<br>❐99 Refusal
2.4 What was your age approximatively when you first used it?	_____<br>❐88 DNK<br>❐99 Refusal	_____<br>❐88 DNK<br>❐99 Refusal	_____<br>❐88 DNK<br>❐99 Refusal	_____<br>❐88 DNK<br>❐99 Refusal	_____<br>❐88 DNK<br>❐99 Refusal	_____<br>❐88 DNK<br>❐99 Refusal	_____<br>❐88 DNK<br>❐99 Refusal	_____<br>❐88 DNK<br>❐99 Refusal	_____<br>❐88 DNK<br>❐99 Refusal	_____<br>❐88 DNK<br>❐99 Refusal	_____<br>❐88 DNK<br>❐99 Refusal	_____<br>❐88 DNK<br>❐99 Refusal
2.5 How much did you spend in the <u>last week</u> to purchase this drug?	_____<br>Baht<br>❐ Free<br>❐88 DNK<br>❐99 Refusal	_____<br>Baht<br>❐ Free<br>❐88 DNK<br>❐99 Refusal	_____<br>Baht<br>❐ Free<br>❐88 DNK<br>❐99 Refusal	_____<br>Baht<br>❐ Free<br>❐88 DNK<br>❐99 Refusal	_____<br>Baht<br>❐ Free<br>❐88 DNK<br>❐99 Refusal	_____<br>Baht<br>❐ Free<br>❐88 DNK<br>❐99 Refusal	_____<br>Baht<br>❐ Free<br>❐88 DNK<br>❐99 Refusal	_____<br>Baht<br>❐ Free<br>❐88 DNK<br>❐99 Refusal	_____<br>Baht<br>❐ Free<br>❐88 DNK<br>❐99 Refusal	_____<br>Baht<br>❐ Free<br>❐88 DNK<br>❐99 Refusal	_____<br>Baht<br>❐ Free<br>❐88 DNK<br>❐99 Refusal	_____<br>Baht<br>❐ Free<br>❐88 DNK<br>❐99 Refusal

SECTION 3 INJECTION DRUG USE		
"The next questions are related to your injecting practices."		
3.1	How old were you when you injected any drug for the first time?	I__I__I years old<br>❒88 DNK ❒99 Refusal
3.2	When was the last time you injected any drugs?	❒1 Today/yesterday<br>❒2 In last week<br>❒3 In last month (but more than a week ago)<br>❒4 2-6 months ago → 3.4<br>❒88 DNK ❒99 Refusal
3.3	How often did you inject any drugs during the past month?	❒1 Every day<br>❒2 Every second day<br>❒3 Every third day<br>❒4 Once in a week<br>❒5 Once in a month<br>❒88 DNK ❒99 Refusal
3.4	In the last year where or from whom did you get needles/syringes for injection of drugs? ***(Several answers possible)***	❒1 Purchased at a pharmacy<br>❒2 In NGO which has a needles and syringes exchange programme (Specify): ___________________________________<br>❒3 From friends who inject drugs<br>❒4 From friends who do not inject drugs<br>❒5 From family<br>❒77 Other (Specify): ________________________________<br>____<br>____<br>____<br>____<br>____<br>____<br>____<br>❒88 DNK ❒99 Refusal
3.5	Have you ever shared your syringe with other persons?	❒1 No → 3.9<br>❒2 Yes<br>❒88 DNK ❒99 Refusal
3.6	In the last 6 months, how often have you shared your syringe?	❒1 All the time<br>❒2 Almost all the time<br>❒3 Sometimes<br>❒4 Almost never<br>❒5 Never → 3.9<br>❒88 DNK ❒99 Refusal
3.7	Do you usually clean your syringes before sharing them?	❒1 No → 3.9<br>❒2 Yes<br>❒88 DNK ❒99 Refusal

3.8	Which products do you use to clean your syringes when you share them?<br>***(Several answers possible)***	❒1 Water<br>❒2 Bleach<br>❒3 Boiling<br>❒4 Disinfectants<br>❒5 Burning<br>❒77 Other (Specify): ___________________________________<br>____<br>____<br>____<br>*❒88 DNK ❒99 Refusal*
3.9	Have you <u>ever</u> shared your injecting material (containers, water, filter…) with other persons?	❒1 No ➔ 3.11<br>❒2 Yes<br>*❒88 DNK ❒99 Refusal*
3.10	In the last 6 months, how often have you shared your injecting material (containers, water, filter…)?	❒1 All the time<br>❒2 Almost all the time<br>❒3 Sometimes<br>❒4 Almost never<br>❒5 Never<br>*❒88 DNK ❒99 Refusal*
3.11	Have you <u>ever</u> reused your own syringe?	❒1 No ➔ 3.15<br>❒2 Yes<br>*❒88 DNK ❒99 Refusal*
3.12	<u>In the last 6 months</u>, how often did you reuse your own syringe?	❒1 All the time<br>❒2 Almost all the time<br>❒3 Sometimes<br>❒4 Almost never<br>❒5 Never ➔ 3.15<br>*❒88 DNK ❒99 Refusal*
3.13	Do you usually clean your syringes before reusing them?	❒1 No ➔ 3.15<br>❒2 Yes<br>*❒88 DNK ❒99 Refusal*
3.14	Which products do you use to clean your syringes when you reuse them?<br>***(Several answers possible)***	❒1 Water<br>❒2 Bleach<br>❒3 Boiling<br>❒4 Disinfectants<br>❒5 Burning<br>❒77 Other (Specify): ___________________________________<br>____<br>____<br>____<br>*❒88 DNK ❒99 Refusal*

3.15	The last time you injected drugs, did you use a new needle and syringe?	❒1 No<br>❒2 Yes<br>❒3 I don't remember<br>*❒88 DNK ❒99 Refusal*
3.16	Have you ever experienced an overdose (exceeding the limit of the usual drug amount)?	❒1 No<br>❒2 Yes<br>*❒88 DNK ❒99 Refusal*

**SECTION 4 SEXUAL RISK BEHAVIORS AND OTHER RISK FACTORS**		
"Now I am going to ask you some questions about your sexual behavior and tattoo experience. I remind you again that all your responses are completely confidential, and that you can ask to skip a question if you prefer not to answer."		
4.1	Have you ever had sex?	❒1 No ➔ 4.6<br>❒2 Yes<br>*❒88 DNK ❒99 Refusal*
4.2	Have you ever received money in exchange of sex?	❒1 No ➔ 4.4<br>❒2 Yes<br>*❒88 DNK ❒99 Refusal*
4.3	Is sex work your main source of income?	❒1 No<br>❒2 Yes<br>*❒88 DNK ❒99 Refusal*
4.4	Have you ever received drug in exchange of sex?	❒1 No<br>❒2 Yes<br>*❒88 DNK ❒99 Refusal*
4.5	Have you ever had sex with a same-sex partner?	❒1 No<br>❒2 Yes<br>*❒88 DNK ❒99 Refusal*
4.6	Do you have any tattoo or piercing?	❒1 No ➔ Section 5<br>❒2 Yes<br>*❒88 DNK ❒99 Refusal*
4.7	Where did you do it?<br>***(Several answers possible)***	❒1 Professional tattoo parlor<br>❒2 Temple<br>❒3 Prison<br>❒4 With friends<br>❒77 Other (Specify): _<br>_ _<br>_ _<br>_ _<br>*❒88 DNK ❒99 Refusal*

SECTION 5 HCV KNOWLEDGE AND TESTING		
"Now, I am going to ask you some questions about hepatitis C infection.<br>If you want to know more about hepatitis C, you can ask questions at the end of the interview and we'll give you a brochure with information on the disease.<br>If you want to be tested for hepatitis C virus infection, we can refer you to a free testing facility at the end of the interview."		
5.1	Have you ever heard of the hepatitis C virus before this interview?	❒1 No ➔ Section 6<br>❒2 Yes<br>*❒88 DNK ❒99 Refusal*
5.2	Can you tell the symptoms of the hepatitis C virus infection?<br>***(Write all the answers in the order they were mentioned)***	______________________________<br>______________________________<br>______________________________<br>______________________________<br>______________________________<br>______________________________<br>*❒88 DNK ❒99 Refusal*
5.3	In your opinion, can people be infected with Hepatitis C without having any symptoms?	❒1 No<br>❒2 Yes<br>*❒88 DNK ❒99 Refusal*
5.4	Can you tell what you think is the main route of transmission of the hepatitis C virus?<br>***(Write all the answers in the order they were mentioned)***	______________________________<br>______________________________<br>______________________________<br>______________________________<br>______________________________<br>______________________________<br>______________________________<br>*❒88 DNK ❒99 Refusal*
5.5	Have you ever had an HCV test?	❒1 No ➔ Section 6<br>❒2 Yes, in the last 12 months<br>❒3 Yes, more than 12 months ago<br>*❒88 DNK ❒99 Refusal*
5.6	Where have you been tested for HCV?	❒1 Napneung project at AMS / CMU<br>❒77 Other (Specify): ____________________<br>______________________________<br>______________________________<br>______________________________<br>*❒88 DNK ❒99 Refusal*
5.7	What was the result of your last test?	❒1 Positive<br>❒2 Negative ➔ Section 6<br>❒3 Do not remember ➔ Section 6<br>❒4 Result not communicated ➔ Section 6<br>*❒88 DNK ❒99 Refusal*

5.8	Were you offered a treatment?	❒1 No<br>❒2 Yes (Specify): ___________________________________<br>____<br>____<br>____<br>*❒88 DNK ❒99 Refusal*

**SECTION 6 ACCESS TO HEALTH CARE**		
"I will now ask you some questions about your access to health care services."		
6.1	Do you have a health insurance?	❒1 No ➔ 6.3<br>❒2 Yes<br>*❒88 DNK ❒99 Refusal*
6.2	What is your health coverage scheme?	❒1 Universal health care<br>❒2 Social Security Scheme<br>❒3 CSMBS<br>❒4 Private insurance<br>❒77 Other (Specify): ___________________________________<br>____<br>____<br>*❒88 DNK ❒99 Refusal*
6.3	Where do you usually go when you have a health issue?	❒1 Do not look for care (Specify why): ____________________<br>____<br>____<br>____<br>❒2 Local health station<br>❒3 Public hospital<br>❒4 Private hospital or clinic<br>❒5 Traditional healer<br>❒77 Other (Specify): ___________________________________<br>____<br>____<br>*❒88 DNK ❒99 Refusal*
6.4	What kind of barriers to access health care have you ever experienced?<br>***(Show the card with the answers, several answers possible)***	❒1 Fear of police<br>❒2 Fear of stigma<br>❒3 Too expensive<br>❒4 Too far<br>❒5 Fear of diagnosis<br>❒6 None<br>❒77 Other (Specify): ___________________________________<br>____<br>____<br>____<br>*❒88 DNK ❒99 Refusal*

6.5	Have you ever been refused medical care?	❒1 No<br>❒2 Yes<br>*❒88 DNK ❒99 Refusal*
6.6	Have you ever experienced discriminatory attitudes in health care settings in relation to your drug use?	❒1 No<br>❒2 Yes (Specify): ________________________________<br>____<br>____<br>____<br>____<br>____<br>*❒88 DNK ❒99 Refusal*
6.7	Have you ever received care for drug addiction problems?	❒1 No ➔ 6.10<br>❒2 Yes<br>*❒88 DNK ❒99 Refusal*
6.8	Are you or have you been on MMT?	❒1 No<br>❒2 Yes (Still receiving MMT: ❒1 No ❒2 Yes)<br>*❒88 DNK ❒99 Refusal*
6.9	Have you ever been on <u>voluntary</u> drug rehabilitation?	❒1 No<br>❒2 Yes (Specify where for each time):<br>1st time: _________________<br>2nd time: _________________<br>3rd time: _________________<br>4th time: _________________<br>*❒88 DNK ❒99 Refusal*
6.10	Are you a member of a support group for drug users?	❒1 No<br>❒2 Yes (Specify): _________________<br>*❒88 DNK ❒99 Refusal*
6.11	Have you ever participated in a health survey targeting drug users?	❒1 No<br>❒2 Yes<br>*❒88 DNK ❒99 Refusal*

**SECTION 7 CONTACT WITH THE POLICE/PRISON/CORRECTION AND SIMILAR FACILITIES**		
"Now I am going to ask you about your contacts with the police and/or other representatives of law."		
7.1	Have you <u>ever</u> been arrested by the police in relation to your drug use?	❒1 No<br>❒2 Yes<br>*❒88 DNK ❒99 Refusal*
7.2	Have you <u>ever</u> been in prison?	❒1 No ➔ 7.6<br>❒2 Yes<br>*❒88 DNK ❒99 Refusal*

7.3	Have you ever used drugs while in prison?	❒1 No ➔ 7.6<br>❒2 Yes<br>*❒88 DNK ❒99 Refusal*
7.4	Have you ever injected drugs while in prison?	❒1 No ➔ 7.6<br>❒2 Yes<br>*❒88 DNK ❒99 Refusal*
7.5	Have you shared syringes with other persons while in prison?	❒1 No<br>❒2 Yes<br>*❒88 DNK ❒99 Refusal*
7.6	Have you ever been in compulsory drug treatment?	❒1 No ➔ Section 8<br>❒2 Yes (Specify how many times): _ _ _ _ _ _ _ _<br>*❒88 DNK ❒99 Refusal*
7.7	What was the compulsory drug treatment? ***(Several answers possible)***	❒1 Inpatient<br>❒2 Outpatient<br>❒3 Military boot camp<br>❒77 Other (Specify): _<br>_ _<br>*❒88 DNK ❒99 Refusal*

**SECTION 8 EXPECTATIONS OF HEALTH CARE SYSTEM**		
8.1	If you need information about health issues, where do you look for them? ***(Show the card with the answers, several answers possible)***	❒1 Ask health care professionals<br>❒2 Ask friends<br>❒3 Search on the internet<br>❒77 Other (Specify): _<br>_ _<br>*❒88 DNK ❒99 Refusal*
8.2	***(Skip this question in case the person already knows that she/he is infected with HCV)*** Would you be interested in knowing your HCV status?	❒1 No (Specify why): _<br>_ _<br>❒2 Yes<br>*❒88 DNK ❒99 Refusal*
8.3	If there was a new HCV treatment available in Thailand, would you like to be informed?	❒1 No ➔ 8.5<br>(Specify why): _<br>_ _<br>❒2 Yes<br>*❒88 DNK ❒99 Refusal*

8.4	What would be the best way to inform you?	❒1 Poster/leaflet in a NGO<br>❒2 Website<br>❒3 Text messages<br>❒4 Telephone call<br>❒77 Other (Specify): _<br>❒88 *DNK* ❒99 *Refusal*
8.5	What suggestions do you have to improve the governmental policies toward people who inject drugs?<br>***(Write down all answers)***	_ _<br>_ _<br>_ _<br>_ _<br>_ _<br>_ _<br>❒88 *DNK* ❒99 *Refusal*

SECTION 9 INTERVIEWEE'S FEEDBACK

9.1	What is your opinion about this interview?	**Usefulness:**<br>❐1 Very useful<br>❐2 Useful<br>❐3 Ok<br>❐4 Not useful<br>❐5 Waste of time<br><br>**Embarrassing:**<br>❐1 Not at all embarrassing<br>❐2 Not embarrassing<br>❐3 Ok<br>❐4 Embarrassing<br>❐5 Very embarrassing<br><br>**Duration:**<br>❐1 Very short<br>❐2 Short<br>❐3 Ok<br>❐4 Long<br>❐5 Very Long

9.2 Do you have any comments about the questionnaire?

_ _
_ _
_ _
_ _
_ _
_ _
_ _
_ _
_ _
_ _
_ _
_ _
_ _
_ _
_ _
_ _
_ _
_ _
_ _
_ _
_ _
_ _
_ _

"THANK YOU A LOT FOR TAKING PART IN THIS STUDY"

**SECTION 10 INTERVIEWER'S EVALUATION**		
To be completed once the interviewee has left the room		
10.1	Time interview end	I__I__I H I__I__I M
10.2	The interview was performed:	❒1 Without any interruption<br>❒2 The person showed signs that he/she wanted to stop the interview<br>❒3 Was interrupted several times
10.3	Describe the person interviewed :<br>***(Can be more than one answer)***	❒1 Has a visible physical problem or handicap<br>❒2 Was relaxed<br>❒3 Was OK<br>❒4 Was very emotional<br>❒5 Was watching at the time<br>❒6 Had difficulty understanding<br>❒7 Had difficulty expressing her/himself<br>❒8 Had difficulty remembering events<br>❒9 Was physically tired
10.4	The answers seemed:	❒1 Very reliable<br>❒2 Reliable<br>❒3 Not reliable<br>❒4 Not at all reliable<br>❒88 Don't know
10.5	Comments:	--<br>--<br>--<br>--<br>--<br>--<br>--<br>--<br>--<br>--<br>--<br>--<br>--<br>--
